# Bronchoscopic Interventional Therapy Combined With Pembrolizumab in the Treatment of Pulmonary Large Cell Neuroendocrine Carcinoma: A Case Report

**DOI:** 10.1111/crj.70009

**Published:** 2024-09-03

**Authors:** Yingyi Fan, Yingying Wang, Yanrong Ji, Shuang Li, Jian Zhang, Xingliang Hao

**Affiliations:** ^1^ Department of Respiratory and Critical Care Medicine Shengli Oilfield Central Hospital Dongying China; ^2^ Clinical Pharmacy of Pharmacy Department Shengli Oilfield Central Hospital Dongying China

**Keywords:** central airway stenosis, large cell neuroendocrine carcinoma of the lung, pembrolizumab, photodynamic therapy

## Abstract

This study reports a significant clinical outcome following the use of bronchoscopic interventional therapy combined with pembrolizumab for treating pulmonary large cell neuroendocrine carcinoma (LCNEC), showcasing a novel approach in managing this aggressive cancer.

## Graphical abstract

Bronchoscopic interventional therapy combined with pembrolizumab in the treatment of Large cell neuroendocrine carcinoma of the lung has achieved good results.

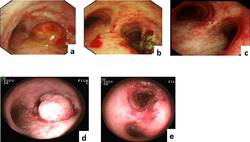

## 1 Introduction

Large‐cell neuroendocrine carcinoma (LCNEC) of the lung is a rare and highly aggressive high‐grade neuroendocrine carcinoma, accounting for approximately 2.1%–3.5% of primary lung tumours. Currently, no established standard treatment exists for LCNEC. Surgery is primarily involved in the early stages, whereas multidisciplinary treatment is often required in the middle and late stages. Herein, we report a case of central airway and bilateral lung metastases that achieved favourable outcomes following bronchoscopic interventional therapy combined with immunotherapy.

## 2 Case Report

**FIGURE 1:**
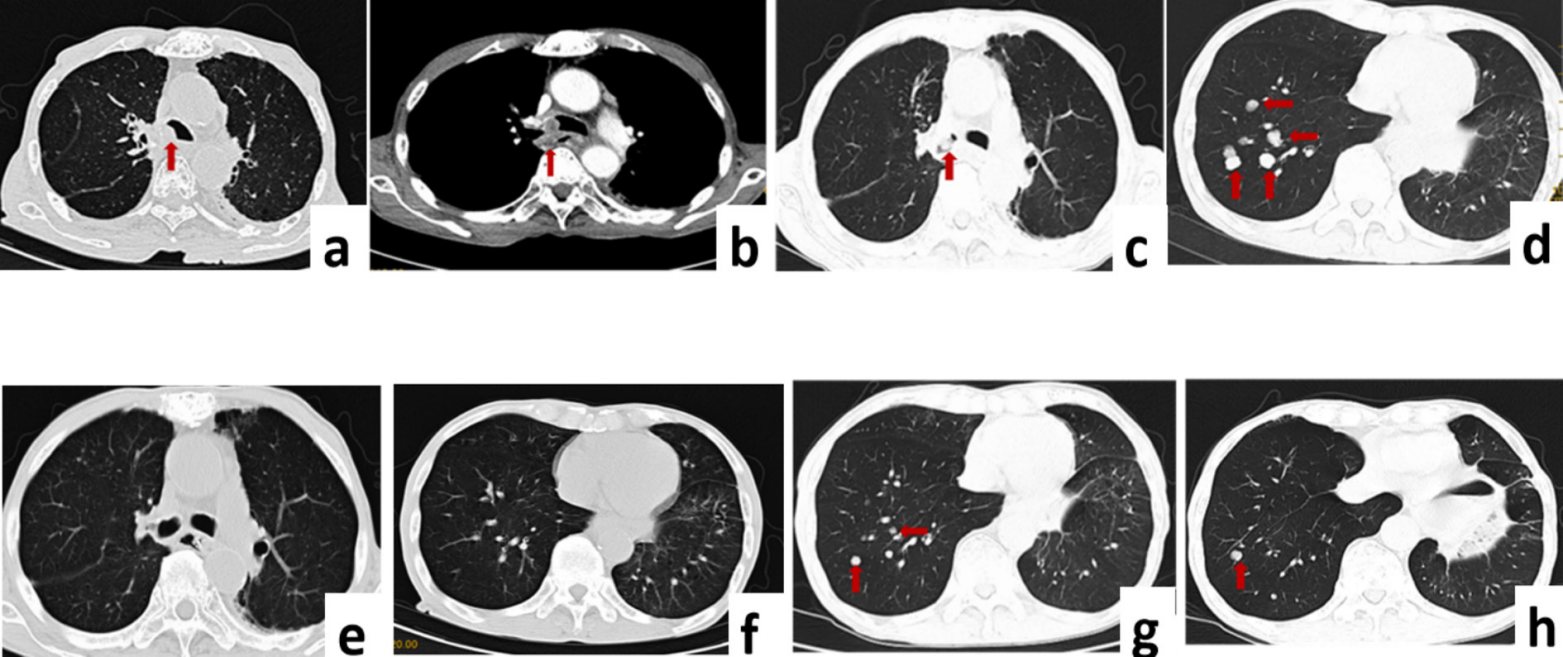
Enhanced CT of the lung. (a, b) Carina and right main bronchus lesions. (c, d) Tumour recurrence in the right main bronchus and multiple metastases in the right lung. (e, f) Complete response of metastatic lesions in the right main bronchus and lung after 6 months of immunotherapy. (g, h) Progression after treatment of lung lesions, new multiple lung metastases.

**FIGURE 2:**
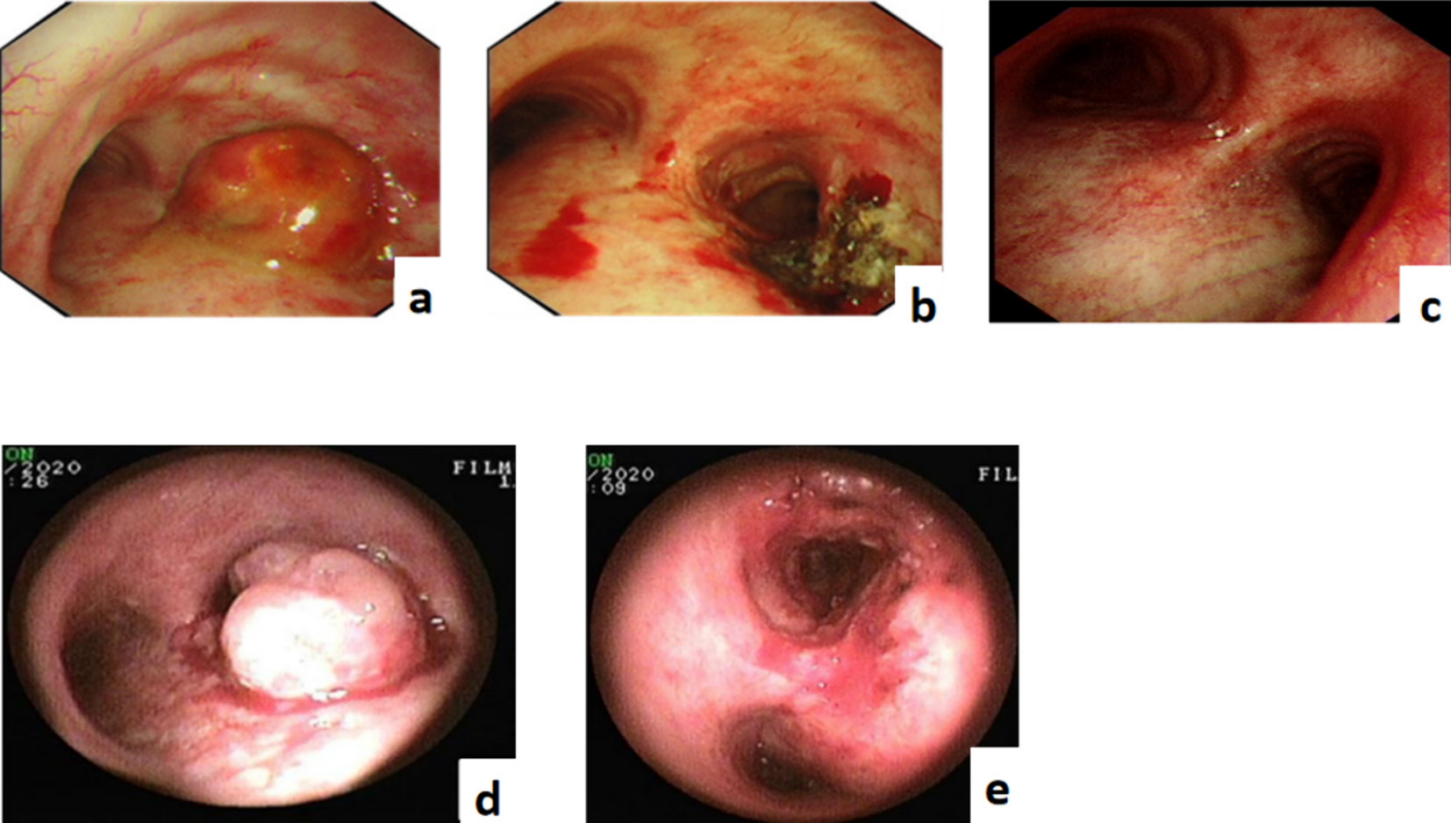
Bronchoscopy. (a, b) Comparison before and after the first bronchoscopic intervention. (c) Complete response of metastatic lesions in the right main bronchus after 6 months of immunotherapy. (d, e) Second bronchoscopic interventional therapy combined with photodynamic therapy.

**FIGURE 3:**
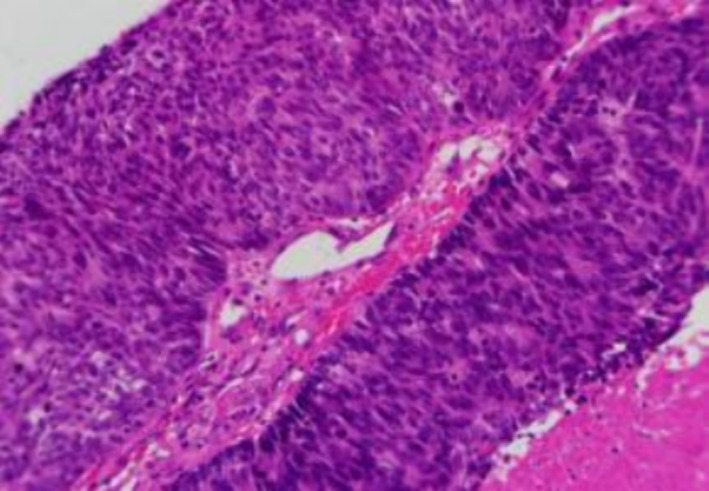
Pathology: Large cell carcinoma.

In May 2019, an 82‐year‐old man presented with a cough with white viscous sputum, chest distress, shortness of breath after activities and a small amount of blood streaks in the sputum. Enhanced computed tomography (CT) of the lung revealed soft tissue shadows at the opening of the right main bronchus (Figure 1a,b). Brain magnetic resonance imaging and bone‐enhanced CT showed no signs of tumour metastasis. Bronchoscopy with endotracheal intubation was performed under general anaesthesia on 6 June 2019. Microscopically, the neoplasm at the carina completely obstructed the right main bronchus, and the tumour surface was rich in blood vessels and bled easily. Ablation with an electric snare, carbon dioxide freezing and argon plasma coagulation (APC) were performed to remove the intraluminal neoplasm. The lesion affected the carina, maintaining patency of the right upper lobe, intermediate bronchus and middle and lower lobes (Figure 2a,b). Postoperative pathology revealed poorly differentiated large cell neuroendocrine carcinoma (dimensions of the greyish‐white tissue submitted: 1.5 × 1.0 × 0.8 cm) (Figure 3). Broad‐spectrum CK showed 3+ intensity; TTF‐1 was scattered positive; NapsinA was negative; Ber‐EP4 was positive; CK5/6, CK7 and p63 were positive; Ki‐67 showed 55%–60% positivity; Syn was 2+; CgA was positive. Disease diagnosis was confirmed as pulmonary large cell neuroendocrine carcinoma, staged at T4N0M0. According to the Lung Cancer Treatment Guidelines, the patient, with a PS score of 3, was deemed unsuitable for chemotherapy; therefore, 50 Gy of local radiation therapy was administered. On 26 August 2019, lung CT scans indicated that the lesions were stable post‐radiotherapy.

In February 2020, the patient developed haemoptysis, chest tightness and shortness of breath. Lung CT was performed on 16 March 2020: there was a possibility of recurrence of the mass at the opening of the right main bronchus after treatment, and multiple nodules in the bilateral lung were considered metastases (Figure 1c,d). On 25 March, bronchoscopy with endotracheal intubation under general anaesthesia was performed, and the right main bronchus was completely obstructed by neoplasms at the carina microscopically. Interventional ablation such as snare, APC and carbon dioxide freezing was performed to remove the tumour in the right main bronchus, which showed lesions involving the carina and right main bronchus, infiltrative changes, obstruction of the right upper lobe and atelectasis (Figure 2d,e). Pathological results indicated large‐cell neuroendocrine carcinoma. *EGFR*, *ALK*, *ROS1* and *TP53* gene mutations were negative, programmed death ligand 1 (PD‐L1) expression was <1%, and the tumour mutational burden (TMB) was 28.3 mut/Mb. The treatment plan was formulated based on multi‐disciplinary therapy by interventional pulmonology specialists, oncologists and imaging physicians. Local lesions were treated with photodynamic therapy (PDT), and pembrolizumab systemic immunotherapy (200 mg every 3 weeks) was administered. Subsequently, lung CT follow‐up was conducted bi‐monthly, demonstrating that the lung metastases progressively diminished. On 5 October 2020, lung CT indicated no recurrence of lesions in the right main bronchus, and the lung metastases had disappeared. Bronchoscopy showed no tumour recurrence in the carina (Figures 1e,f and 2c). There were no signs of tumour metastasis, and the disease was classified as in complete remission (CR).

Thereafter, pembrolizumab maintenance (200 mg every 3 weeks) was continued; on 10 October 2021, lung CT showed multiple intrapulmonary metastases again, indicating disease progression (Figure 1g,h). After the second interventional ablation combined with photodynamic therapy, pembrolizumab immunotherapy was maintained, and progression‐free survival (PFS) reached 18 months.

## 3 Discussion

Pulmonary LCNEC has a high degree of malignancy, strong invasiveness and short median overall survival. Early stage (stages I, II and III) is dominated by surgery, and postoperative adjuvant chemotherapy can benefit survival; stage IV patients with locally advanced unresectable and distant metastasis are dominated by chemotherapy and require multidisciplinary treatment [1]. Malignant central airway lesions are treated using a combination of cryotherapy and APC [2]. PDT employs photosensitizers with a high affinity for tumour tissue, functioning through two mechanisms: free radicals and singlet oxygen, which promote tumour cell apoptosis or death. Furthermore, PDT can generate thromboxane, embolize tumour nutrient vessels and facilitate tumour cell death [3]. Additionally, it may induce anti‐tumour immune responses and augment the efficacy of immune cells against tumours in the body [4].

The patient's lesion caused obstruction of the right main bronchus and cardia involvement, characterized by endogenous stenosis. Rapid airway ablation, employing respiratory endoscopic electric snare, APC, carbon dioxide freezing and other therapies, smoothed the airway. PDT treated residual and surrounding infiltrative lesions, yielding good long‐term outcomes and markedly enhancing prognosis.

A study from the National Cancer Database showed that immunotherapy could improve the survival rate of LCNEC [5]. In a study of 10 patients with advanced LCNEC who progressed after platinum‐based first‐line chemotherapy and were treated with nivolumab or pembrolizumab, 60% achieved a partial response, with a median PFS of 13.3 months [6]. Some studies have indicated that positive expression of PD‐L1 in LCNEC tumour cells is associated with a more favourable prognostic trend and longer overall survival [7]. However, case reports also exist of LCNEC patients who, despite negative PD‐L1 expression by immunohistochemistry, exhibited positive mutations in genes such as RB1 and TP53, along with a high TMB (24.76 mut/Mb). Following one cycle of pembrolizumab treatment, these tumour lesions shrank, and improvement continued over 6 months [8]. TMB is prevalently expressed in patients with pulmonary LCNEC [9]. Currently, prospective randomized controlled trials that support the use of immunotherapy for pulmonary neuroendocrine tumours are lacking. PD‐L1 and TMB are the primary predictive markers, yet their predictive capacity for the efficacy of immune checkpoint inhibitors remains uncertain [10].

*EGFR*, *ALK*, ROS1 and other gene mutations were negative, and immunohistochemical PD‐L1 expression was negative, with a high TMB of 28.3 mut/Mb. After 6 months of pembrolizumab monotherapy, the patient underwent a comprehensive evaluation for CR without noting any immune checkpoint inhibitor‐related adverse reactions. The treatment continued, extending PFS to 18 months and demonstrating significant clinical benefits. Similarly, in an LCNEC case from China, immunohistochemical analysis revealed negative PD‐L1 expression and a high TMB of 25.8 mut/Mb. This patient also showed a durable response to nivolumab, with no tumour recurrence during a 20‐month follow‐up [11]. These findings align with prior reports, suggesting that TMB may serve as a predictor of immunotherapy efficacy in patients with pulmonary LCNEC.

Central pulmonary LCNEC often causes malignant airway stenosis, which is difficult to remove. Nevertheless, bronchoscopic intervention can swiftly ease the airway and reduce obstructive symptoms. Moreover, some patients may benefit from immunotherapy. Tailored multidisciplinary treatment models need further clinical advancement.

## Author Contributions

**Yingyi Fan**: data curation, writing – original draft. **Yingying Wang**: data curation, writing – original draft. **Yanrong Ji**: data curation, writing – review and editing. **Shuang Li**: investigation, writing – review and editing. **Jian Zhang**: writing – review and editing. **Xingliang Hao**: conceptualization, investigation, writing – review and editing.

## Ethics Statement

This study was approved by the Ethics Committee of Shengli Oilfield Central Hospital and was conducted in accordance with the Declaration of Helsinki. Written informed consent was obtained from the patient for the publication of this report in accordance with the journal's patient consent policy.

## Conflicts of Interest

The authors declare no conflicts of interest.

## Data Availability

The data that support the findings of this study are available from the corresponding author upon reasonable request.
